# Rheological Behavior and Aging Resistance of SBS/Lignin Composite Modified Asphalt

**DOI:** 10.3390/polym18111319

**Published:** 2026-05-27

**Authors:** Wenliang Wu, Longfei Li, Mukai Huang, Junxuan Liang, Zhi Li

**Affiliations:** 1 School of Civil Engineering and Transportation, South China University of Technology, Guangdong 510641, Guangzhou, China; ctwlwu@scut.edu.cn (W.W.);; 2 Xin Yue (Guangzhou) Material Technology Research Institute Co., Ltd., Guangdong 510006, Guangzhou, China

**Keywords:** SBS/lignin composite modified asphalt, rheological properties, antioxidative performance, molecular dynamics simulation

## Abstract

The degradation of styrene-butadiene-styrene (SBS) modified asphalt under thermal-oxidative aging can reduce pavement service life. Lignin is a renewable material with active phenolic hydroxyl groups. Incorporating lignin into SBS modified asphalt may provide a potential bio-based auxiliary modification route. To investigate the antioxidative effect and rheological properties of SBS modified asphalt after adding lignin, a molecular dynamics test and experimental tests were employed. The molecular simulation results suggested that lignin preferentially associated with asphaltene and resin molecules and changed the molecular mobility of asphalt components in a component-dependent manner. The SBS/lignin composite modified asphalt was evaluated by temperature sweep (TS), Multiple Stress Creep and Recovery (MSCR), Linear Amplitude Sweep (LAS) and Fourier transform infrared spectroscopy (FTIR). Rheological tests showed that lignin increased the complex shear modulus and rutting factor. LAS results showed that lignin reduced the fatigue life of SBS-modified asphalt in the unaged state due to increased stiffness and embrittlement. However, after long-term aging, the lignin-containing binders retained higher fatigue resistance than the SBS-only control, which may be related to the slower evolution of oxidation-related functional groups and SBS-related spectral indices. FTIR analysis provided semi-quantitative evidence that lignin addition reduced the growth of sulfoxide-related bands and helped maintain the polybutadiene-related index during aging. Overall, lignin may serve as a potential auxiliary antioxidant modifier for SBS modified asphalt, while its exact source-specific molecular mechanism requires further verification.

## 1. Introduction

As a crucial component of global infrastructure, the asphalt manufacturing sector carries a massive economic impact. The global asphalt market was valued at over USD 168 billion in 2024 and is projected to experience continuous growth driven by rapid urbanization and escalating road construction investments worldwide [1]. In the field of styrene-butadiene-styrene (SBS) asphalt, the aging resistance and rheological properties of asphalt materials are key factors governing service life [2,3,4]. With the constant increase in traffic loading and the developing frequency of extreme climatic conditions, SBS modified asphalt can no longer fully satisfy the performance requirements of modern pavements [5,6]. Lignin, a naturally occurring organic macromolecule with reserves second only to cellulose, has attracted increasing attention [7,8]. Because of its abundant phenolic hydroxyl groups, lignin has been reported to exhibit a certain resistance to thermal-oxidative aging [9,10,11]. Compared with some conventional antioxidants, lignin has been reported to have potential material-level advantages, such as renewability, wide availability, and biodegradability [12,13,14]. However, the chemical properties and self-aggregation behavior of lignin are strongly dependent on its extraction process and molecular structure. Wibowo et al. demonstrated that lignin obtained by different methods (e.g., milled wood lignin, organosolv lignin, kraft lignin) possess distinct molecular conformations, hydroxyl group distributions, and morphological characteristics, which directly affect their interfacial interactions with other materials [15]. Marcuello et al. showed that the nanoscale structuration of lignin—its functional group arrangement and spatial organization—governs its chemical affinity and adhesive properties, influencing the compatibility with polymer matrices [16]. These findings highlight that the source and processing history of lignin must be taken into account when interpreting its performance as an asphalt modifier. Owing to the structural similarity between lignin molecules and the aromatic components in asphalt, lignin has been shown to enhance both rutting and oxidation resistance of asphalt binders [17,18,19]. Vaidya et al. incorporated different proportions of soda lignin powder into asphalt and found that both viscosity and rheological performance were improved [20]. Norgbey et al. introduced lignin fibers into asphalt and, based on DSR results, reported enhanced rutting resistance, improved recovery behavior, and better adhesion to aggregates in lignin-modified asphalt [12]. Using ReaxFF reactive simulations, Huang et al. found that cellulose and hemicellulose components in lignin did not contribute to antioxidative performance, and that excessive lignin content could even be detrimental to asphalt performance [21]. Through LAS tests, Xu et al. showed that the fatigue life of asphalt modified with 5% lignin decreased, whereas a lignin content of 10% appeared to yield some improvement [22]. These studies collectively suggest that lignin can act as an antioxidant additive in asphalt and thereby improve its high-temperature oxidation resistance and rheological properties. However, lignin incorporation restricts molecular diffusion in asphalt, which may have an adverse effect on fatigue resistance.

In recent years, advances in computational technology have promoted the application of molecular simulation methods in asphalt research. In 2007, Zhang and Greenfield at the University of Rhode Island first introduced molecular dynamics (MD) simulation into asphalt materials research to analyze physical properties such as density and bulk modulus [23]. In 2014, Li and Greenfield further proposed the four-component, twelve-molecule asphalt model, marking the formal adoption of molecular simulation for asphalt performance investigation [24]. Therefore, applying molecular dynamics simulation to the study of modified asphalt can help deepen the understanding of its underlying mechanisms. Compared with lignin, SBS has long been used as a modifier in asphalt [25]. SBS modified asphalt has been widely used in pavement engineering due to its excellent high- and low-temperature performance [26]. However, numerous studies have shown that the polybutadiene (PB) segments in SBS, which contain unsaturated double bonds, are highly susceptible to chain scission or crosslinking reactions under thermal-oxidative conditions [27]. This can damage the elastic network formed by SBS and consequently deteriorate the performance of modified asphalt [28,29,30]. Given the antioxidative potential of lignin, the incorporation of lignin into SBS modified asphalt to form an SBS/lignin composite asphalt may not only compensate for the poor low-temperature performance associated with lignin only modification, but also improve the oxidation resistance of the modified asphalt under complex service environments. Existing studies have mainly focused on lignin as a single modifier, whereas research on SBS/lignin composite modification remains limited. For example, Xiao et al. separately incorporated enzymatic lignin and alkali lignin into an SBS elastic matrix after treatment, and the results showed antioxidative performance comparable to that of Irganox 1010 [31]. In addition, recent work on recycled and bio-based construction materials indicates that sustainability claims should be supported by life-cycle, cost, durability, and service-scale evaluations [32,33]. Therefore, the present study is positioned as a binder-scale investigation of SBS/lignin composite modified asphalt, aiming to clarify its rheological response, aging-related spectral evolution, and possible molecular interaction tendencies, while recognizing that morphology, mixture performance, field durability, and life-cycle benefits require further verification.

In this study, experimental testing was combined with molecular simulation to explore the composite modification of asphalt by SBS and lignin. The main objective of this study is to evaluate the rheological and aging-related responses of SBS/lignin composite modified asphalt within a selected dosage matrix, and to identify a relatively favorable formulation within this investigated range.

## 2. Materials

### 2.1. Preparation of Experimental Materials

The primary materials used in this study included base asphalt, styrene-butadiene-styrene (SBS) modifier, and lignin. Their basic properties are summarized as follows.

Base asphalt: We used 70# petroleum road asphalt as the base asphalt. Its basic technical properties met all the specification requirements. Table 1 gives some typical physical properties of this base asphalt.

SBS modifier: the SBS used in this study was obtained from Yueyang Baling Petrochemical Co., Ltd. (Yueyang, China), with the specific product designation YH-792, the technical specifications are presented in Table 2.

Lignin: the functionality and processing routes of lignin strongly affect its antioxidant activity [34]. To ensure that the molecular weight of lignin is as small as possible and that the phenolic hydroxyl groups are functional, this study used lignin after biological enzymatic hydrolysis, referred to as enzymatic hydrolysis lignin. We used enzymatic hydrolysis lignin from corncob, which had a purity of 70%, the technical specifications are presented in Table 3. Before use, the lignin was dried at 105 °C for several hours to remove moisture and reduce particle agglomeration during subsequent mixing.

### 2.2. Preparation of SBS/Lignin Composite Modified Asphalt

To prepare the SBS/lignin composite modified asphalt, we used a high-temperature shear mixing approach. The first step was to heat the base asphalt in an oven at 135 °C until it had completely melted. It was then transferred to an oil bath maintained at 180 °C. According to the designed proportions, the SBS modifier was gradually added and mixed at a low speed of 600 r/min for approximately 15 min to allow the SBS to disperse within the asphalt. This was followed by high-speed shearing at 5000 r/min for 40 min to ensure sufficient swelling of the SBS. After the SBS had been uniformly incorporated into the asphalt, the pre-dried lignin powder was slowly added under stirring, and the mixture was further sheared at approximately 5000 r/min for another 40 min to ensure thorough dispersion of lignin in the asphalt matrix. After mixing, the prepared composite modified asphalt was allowed to stand at 180 °C for about 2 h to eliminate bubbles and internal stresses introduced during the shearing process.

As a typical thermoplastic elastomer modifier, the dispersion state of SBS in asphalt is closely related to its content. Research by Dong et al. indicates that at low SBS contents, the polymer is primarily dispersed as small particles within the continuous asphalt phase; as the SBS content increases, the particle size distribution gradually broadens, and the system progressively develops into a polymer network structure [35]. However, when the SBS content is further increased to higher levels, the limited light components in the asphalt struggle to fully swell the SBS, which can easily lead to a complex particle distribution, reduced compatibility and hardening of the matrix [36]. Consequently, SBS modified asphalt typically has a suitable content range that balances dispersion, network formation and engineering performance. The study also noted that the maximum SBS content in industrial applications is typically around 3–4%, and that the optimal polymer content should be determined based on the formation of a critical network structure between the asphalt and the polymer. Therefore, selecting an SBS content of 3–4% allows for the investigation of the synergistic effects of lignin on different SBS network states. Research by Gao et al. indicates that lignin can improve the viscosity, elastic modulus and rutting resistance of bitumen; however, the fatigue life of lignin-modified bitumen is reduced compared to that of the base bitumen, with this reduction being more developed at higher loading levels [37].

**Figure 1:**
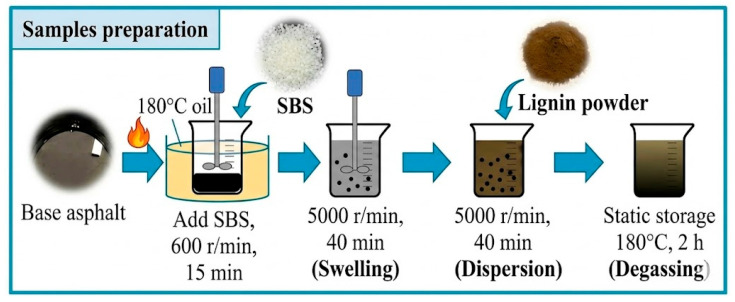
Sample preparation procedure.

Based on the above findings, this study employed a combination of 3–4% SBS and 1–3% lignin. On the one hand, in the 3% SBS system, SBS is still primarily in the stage of transitioning from a dispersed phase to a network phase; an appropriate amount of lignin can improve the insufficient network structure through particle filling and rigid support, but an excessive amount of lignin may compete with SBS for components in the asphalt, thereby affecting the swelling and dispersion of SBS. On the other hand, in the 4% SBS system, the SBS network structure is expected to be more developed. The addition of lignin may further reinforce the binder through a particle-filling effect and possible interactions with polar asphalt fractions, thereby improving high-temperature stability and aging-related resistance, however, if the lignin content is too high, it will lead to excessive stiffness in the system and have an adverse effect on low-temperature ductility and fatigue performance. Therefore, controlling the SBS content at 3–4% and the lignin content at 1–3% not only covers the critical transition range where the SBS network structure evolves from underdeveloped to relatively well-developed, but also allows for the investigation of the synergistic effects between low-level lignin-based filler reinforcement and moderate-level antioxidant properties, whilst avoiding the deterioration in compatibility, increased application viscosity and loss of low-temperature performance caused by excessive modifier content. It should be noted that this formulation design was intended to compare the effect of lignin within a selected SBS-modified asphalt matrix. The SBS dosage was limited to 3% and 4%, and the lignin dosage was limited to 1–3%. In addition, lignin-only modified asphalt was not included as a separate control group. Therefore, the present design is suitable for identifying performance trends within the selected SBS/lignin matrix, but it is not sufficient to establish a general formulation rule or to isolate the independent effect of lignin without SBS. The overall preparation procedure is illustrated in Figure 1, and the final sample designations are listed in Table 4.

## 3. Methods

### 3.1. Molecular Simulation Methodology

**Figure 2:**
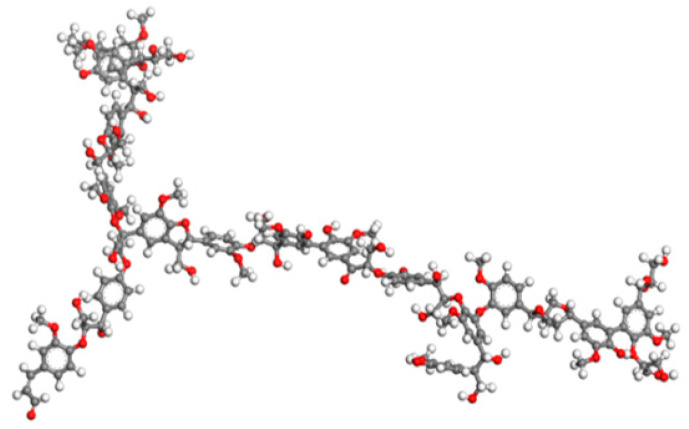
Adler lignin model. In the 3D structures, the atoms are color-coded as follows: grey for Carbon (C), white for Hydrogen (H), red for Oxygen (O). The same applies to the figures below.

Based on previous studies on lignin, several molecular models have been proposed to represent lignin structure. In this study, Adler softwood lignin was selected to represent the lignin molecule because it is one of the most widely accepted models in the literature [38]. Furthermore, it contains typical phenylpropane units, common lignin linkages, and oxygen-containing functional groups such as hydroxyl, methoxy, carbonyl, and aldehyde groups. These structural features are relevant to the polarity and intermolecular interactions of lignin in asphalt systems. However, this model is not intended to reproduce the exact molecular composition of the corncob enzymatic hydrolysis lignin used in the laboratory, which contains lignin together with cellulose, hemicellulose, ash, and other source-dependent constituents. Therefore, the simulation results were interpreted qualitatively and were used only to support possible interaction tendencies among lignin, SBS, and asphalt fractions, rather than to confirm the exact source-specific chemistry of the experimental lignin. Its specific molecular structure is shown in Figure 2.

**Figure 3:**
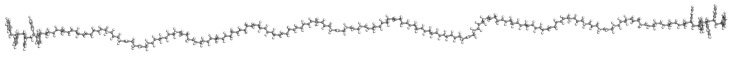
SBS model.

As a modifier, styrene-butadiene-styrene (SBS) is mainly composed of polystyrene (PS) and polybutadiene (PB) segments. The SBS used in this study had a linear structure, with a commonly adopted styrene-to-butadiene ratio of 3:7 [39]. Its molecular structure is presented in Figure 3.

**Figure 4:**
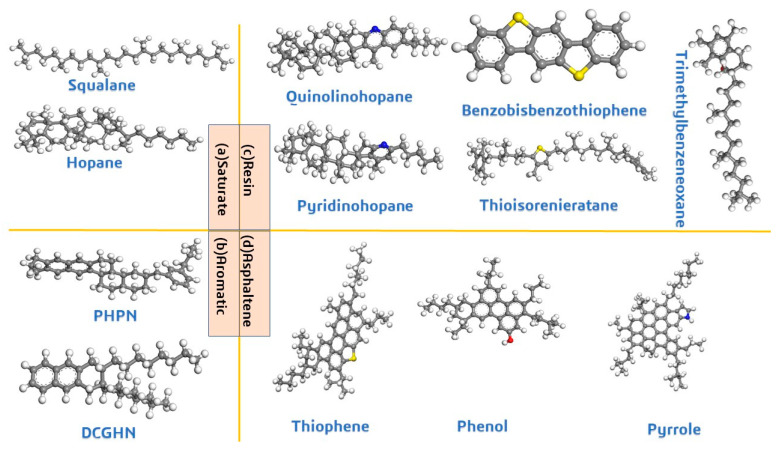
Asphalt model. In the 3D structures, the atoms are color-coded as follows: grey for Carbon (C), white for Hydrogen (H), yellow for Sulfur (S), red for Oxygen (O), and blue for Nitrogen (N).

In molecular dynamics studies of asphalt, the twelve-component asphalt model is commonly used, among which the twelve-molecule asphalt model proposed by Li and Greenfield remains the most widely applied [24]. Accordingly, this model was adopted in this study, and the corresponding molecular structures are shown in Figure 4.

Molecular dynamics simulations were carried out using Materials Studio (v2023). The COMPASS III force field was selected, and the twelve-molecule asphalt model, lignin model, and SBS model were constructed in Amorphous Cell. Two systems were considered, namely the binary SBS/asphalt system and the ternary SBS/lignin/asphalt system. The molecular formulas and the number of molecules used in the simulations are listed in Table 5. The equilibrated box length was 48.42 Å for the SBS/asphalt system and 49.09 Å for the SBS/lignin/asphalt system.

**Figure 5:**
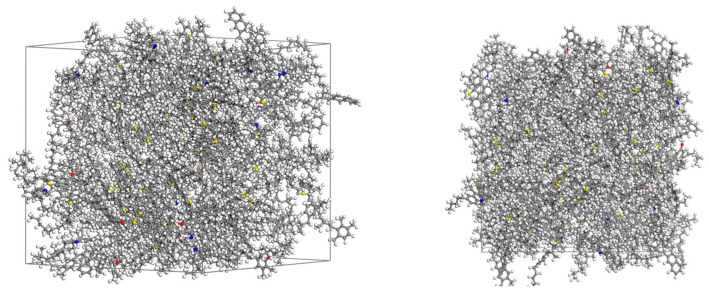
SBS asphalt model. In the 3D structures, the atoms are color-coded as follows: grey for Carbon (C), white for Hydrogen (H), yellow for Sulfur (S), red for Oxygen (O), and blue for Nitrogen (N).

**Figure 6:**
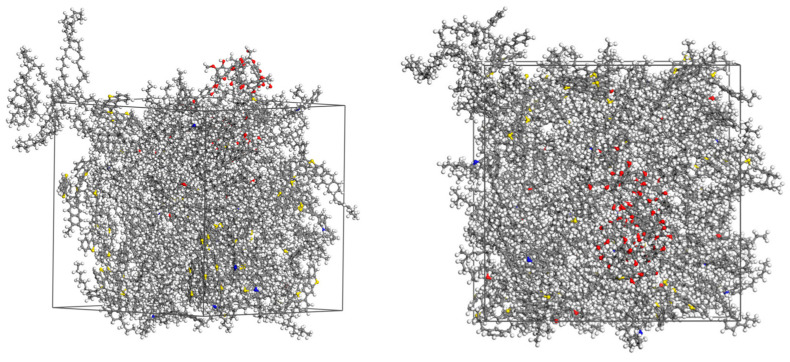
SBS/lignin modified asphalt model. In the 3D structures, the atoms are color-coded as follows: grey for Carbon (C), white for Hydrogen (H), yellow for Sulfur (S), red for Oxygen (O), and blue for Nitrogen (N).

The initial structures were first subjected to geometry optimization to eliminate unreasonable atomic overlaps. This was followed by annealing for 10 cycles between 298 and 498 K to sufficiently sample the configurational space. The time step was set to 1 fs for the molecular dynamics simulations. Subsequently, the systems were equilibrated for 500 ps in the NVT ensemble at 293 K, followed by 500 ps in the NPT ensemble at 293 K to stabilize the density and other system properties. The equilibrated binary and ternary models are shown in Figure 5 and Figure 6.

After obtaining the equilibrated structures and molecular trajectories, the following microscopic indicators were calculated to characterize the system:

RDF: the peak position and intensity of the radial distribution function (RDF) curve can be used to evaluate the aggregation behavior and miscibility of different components at the nanoscale. A smaller peak position and a higher peak intensity indicate a greater relative enrichment of two types of molecules at a given distance. The corresponding equation is given in Equation (1). In this study, particular attention was paid to the RDFs between lignin and the four asphalt fractions in order to analyze the interactions between lignin and each asphalt component.

(1) $$g(r)=\frac{dN}{\rho 4\pi r^{2}dr}$$

where: *g*(*r*) is the radial distribution function; *dN* is the number of targeted particles found within a spherical shell of thickness *dr* at a distance *r*; *ρ* is the average number density of the system; *r* is the radial distance from the reference particle (Å).

Diffusion coefficient (*D*): according to the Einstein diffusion equation, the diffusion coefficient is determined from the slope of the mean square displacement (MSD) of the molecular centroid as a function of time. The expression for the slope of the MSD curve is given as follows:

(2) $$\mathrm{MSD}(t)=\langle {\left|r_{i}(t)-r_{i}(0)\right|}^{2}\rangle$$

(3) $$D=\frac{1}{6}\underset{t\to \infty }{\lim}\frac{d}{dt}MSD(t)$$

where *r_i_*(*t*) and *r_i_*(0) are the position vectors of molecule *i* at time *t* and initial time 0, respectively; *D* is the diffusion coefficient; *t* is the simulation time.

Because only one representative SBS/asphalt binary system and one SBS/lignin/asphalt ternary system were simulated, the MD results were interpreted as qualitative indicators of molecular packing, local association, and component-dependent mobility. The density, MSD, and RDF results were not used to determine phase distribution, dispersion stability, or a complete composite morphology. Such morphology-related conclusions would require independent microscopic validation, such as fluorescence microscopy, AFM phase imaging, or storage stability testing.

### 3.2. Aging Procedures

To examine how lignin influences the aging resistance of asphalt, the prepared composite modified asphalt samples were subjected to two laboratory aging procedures: the Rolling Thin Film Oven (RTFO) for short-term aging and the Pressure Aging Vessel (PAV) test for long-term aging. Short-term aging was conducted using the Rolling Thin Film Oven (RTFO) in accordance with ASTM D2872. For the RTFO procedure, approximately 35 ± 0.5 g of asphalt binder was poured into an aging bottle with a height of 140 mm and a diameter of 64 mm. The oven temperature was maintained at 163 ± 0.5 °C, and hot air was continuously supplied at a flow rate of 4000 ± 200 mL/min. During aging, the bottles were rotated at 15 r/min for 85 min to simulate the thermal-oxidative aging that occurs during asphalt mixing and construction. The obtained short-term aged samples were denoted as xSyL_R, where x and y represent the mass percentages of SBS and lignin in the composite modified asphalt, respectively.

The PAV aging procedure was conducted to simulate the long-term oxidative aging of asphalt during pavement service. Before PAV aging, all binders were first aged by the RTFO procedure. Then, 50 ± 0.5 g of the RTFO-aged binder was placed in an aging pan. After the binder was evenly leveled, the pan was transferred into the pressure aging vessel and aged at 100 °C under 2.1 MPa air pressure for 20 h. According to ASTM D6521, the PAV aging temperature shall be selected to match the climatic conditions of the target region. A temperature of 100 °C corresponds to pavement design temperatures exceeding 64 °C and is appropriate for the tropical and subtropical climate typical of southern China, which is the intended service environment in this study. The resulting long-term aged samples were labeled as xSyL_P, where x and y denote the mass fractions of SBS and lignin, respectively. This aging design allowed the properties of the virgin, short-term aged, and long-term aged binders to be compared systematically.

### 3.3. Temperature Sweep Test

The temperature sweep test was performed using a dynamic shear rheometer (DSR) to evaluate the high-temperature rheological behavior of the SBS/lignin composite modified asphalt. Previous studies have shown that enzymatic hydrolysis lignin modified asphalt and SBS modified asphalt generally fall within the high-temperature PG range of approximately 64–76 °C [40]. Therefore, the test temperature range in this study was set from 40 °C to 88 °C to cover the main service temperature region and the possible high-temperature grade range of the modified binders.

During the temperature sweep test, the loading frequency was fixed at 1.592 Hz, corresponding to an angular frequency of 10 rad/s. The test was conducted for PG-related high-temperature evaluation of the asphalt binders. Different strain amplitudes were applied according to the aging state of the binder. In accordance with AASHTO T 315, a strain amplitude of 12% was used for the virgin samples, while 10% strain was used for the short-term aged samples. Therefore, the strain levels used in this study were selected based on the standard target values for PG-related DSR testing, rather than by independently determining the LVE boundary of each binder through separate strain-amplitude sweep tests. The complex shear modulus G* and phase angle δ were recorded at each temperature. These two fundamental rheological parameters were determined from the maximum applied shear stress and the maximum resulting shear strain. The corresponding equations are defined as follows:

(4) $$G^{*}=\frac{\tau_{\max}}{\gamma_{\max}}$$

(5) δ=ω×Δt

(6) $$RuttingFactor=\frac{G^{*}}{\sin\delta }$$

where *τ*_max_ is the maximum absolute value of the applied shear stress; *γ*_max_ is the maximum absolute value of the resulting shear strain; ϖ is the angular frequency of the oscillation; *δ* is the time lag between the applied stress and the corresponding strain response signals; G* is the complex shear modulus, representing the binder’s total overall resistance to deformation under shear loading; *δ* is the phase angle, reflecting the viscoelastic balance of the material.

By comparing the changes among different formulations, the influence of lignin on the high-temperature stability of SBS modified asphalt was analyzed.

### 3.4. Linear Amplitude Sweep Test

The fatigue behavior of the asphalt binders was characterized using the Linear Amplitude Sweep (LAS) test. According to AASHTO TP101, the test was carried out at 25 °C for both virgin and aged binders. The procedure consisted of two stages. A preliminary frequency sweep at a low strain level was first performed to determine the linear viscoelastic response of the binder. Then, an amplitude sweep was applied at 10 Hz, with the strain gradually increasing from 0.1% to 30% in 1% intervals until failure occurred. During loading, the reduction in storage modulus was recorded to describe the damage evolution of the material. The fatigue life *N_f_* was obtained using the viscoelastic continuum damage approach. The fatigue life curve intuitively characterizes the number of loading cycles that an asphalt binder can withstand up to failure under a given load level. Specifically, the fatigue life is calculated using Equation (7).

(7) $$N_{f}=A{\left(\gamma \right)}^{-B}$$

where *N_f_* is the fatigue life, representing the number of loading cycles to failure; *γ* is the applied shear strain; *A* is the initial fatigue life parameter; *B* is the temperature susceptibility coefficient.

The calculated fatigue lives were then compared among binders with different SBS and lignin dosages to evaluate the effect of lignin on the fatigue resistance of SBS modified asphalt.

### 3.5. Multiple Stress Creep Recovery Test

The Multiple Stress Creep Recovery test was performed to evaluate the high-temperature resistance of asphalt binders to permanent deformation. In accordance with ASTM D7405, both virgin and aged binders were subjected to cyclic loading at 70 °C under two stress levels, namely 0.1 kPa and 3.2 kPa. Before the formal MSCR tests, preliminary experiments were conducted to verify the stability of the testing procedure and parameter settings. The test process was further checked with reference to recent studies on lignin-modified asphalt binders. For the formal MSCR measurements, two parallel specimens were tested for each binder formulation, and the average values were used for the final MSCR curves and comparative analysis. This procedure was adopted to reduce random measurement fluctuation and improve the robustness of the formulation-dependent comparison. During the test, the instantaneous strain under each loading cycle and the residual deformation after unloading were recorded. Based on these responses, the non-recoverable creep compliance (J_nr_) and recovery rate (R) were calculated. Among these parameters, J_nr_ represents the proportion of permanent strain that cannot be recovered under a given stress level, with a lower value indicating better resistance to permanent deformation. In contrast, R is defined as the percentage of strain recovered after unloading relative to the total strain, and a higher value indicates better elastic recovery. In addition to the 3.2 kPa data, stress sensitivity (Jnr-diff) was evaluated using the 0.1 kPa results. All tested binders showed J_nr−diff_ values lower than 75%, indicating that the stress sensitivity of the SBS/lignin composite modified asphalt remained within the commonly accepted MSCR specification limit. By comparing the J_nr_ and R values of modified asphalt binders with different lignin contents in both virgin and aged states, the influence of lignin on the high-temperature deformation resistance of asphalt and its aging-related evolution can be evaluated.

### 3.6. Bending Beam Rheometer Test

The low-temperature rheological properties of the SBS/lignin composite modified asphalt binders were evaluated using a bending beam rheometer (BBR). Both unaged and PAV-aged binders were tested at −6 °C, −12 °C, and −18 °C. Before testing, each asphalt beam specimen was conditioned at the target temperature to ensure thermal equilibrium. During the test, a constant load was applied to the midpoint of the asphalt beam, and the time-dependent deflection was recorded.

The creep stiffness, *S(t)*, was calculated as follows:

(8) $$S(t)=\frac{PL^{3}}{4bh^{3}\delta (t)}$$

where *S(t)* is the creep stiffness at loading time *t*, *P* is the applied load, *L* is the span length, *b* is the specimen width, *h* is the specimen thickness, and *δ(t)* is the mid-span deflection at time *t*.

The creep rate, namely the m-value, was obtained from the slope of the logarithmic stiffness–time curve:

(9) $$m(t)=-\frac{d\log S(t)}{d\log t}$$

where *m(t)* represents the stress relaxation ability of the asphalt binder. A lower *S(t)* indicates better resistance to low-temperature cracking, whereas a higher m-value indicates stronger stress relaxation capacity.

### 3.7. Fourier Transform Infrared Spectroscopy Test

As a commonly used tool for chemical characterization, FTIR testing offers a variety of methods. In this study, the potassium bromide (KBr) pellet method was employed for FTIR analysis. Before testing, the potassium bromide (KBr) particles were thoroughly dried. The presence of moisture can severely interfere with the spectral curve in the range of 4000 cm^−1^ to 3000 cm^−1^ and may reduce the signal-to-noise ratio of the measurement. The FTIR sample was prepared by dissolving the asphalt binder in tetrahydrofuran (THF) at a concentration of 2 mg/mL. Before starting the test, the KBr particles were thoroughly ground to reduce refractive loss of the incident light and improve measurement accuracy. A KBr pellet was pressed using a tablet press, and 1 mL of the asphalt-dissolved THF solution was dropped onto the pellet. The sample was immediately transferred to a 60 °C oven and dried for 5 min to obtain the FTIR scanning specimen. FTIR scans were performed in absorption mode over a range of 4000 cm^−1^ to 400 cm^−1^ at a resolution of 4 cm^−1^. Each sample was scanned 32 times to obtain an average spectrum, and the test temperature was kept constant at 25 °C. After scanning, atmospheric/water vapor compensation was applied. The spectral curves were then baseline-corrected, smoothed, and normalized to obtain the FTIR absorbance curves for all samples. For quantitative analysis, the characteristic peak areas were calculated by integration after baseline correction. The same integration boundaries were used for all samples to ensure comparability. The sulfoxide-related bands were integrated at 1015–1045 cm^−1^ and 1055–1085 cm^−1^, corresponding to the absorption regions around 1030 cm^−1^ and 1070 cm^−1^, respectively. The polybutadiene band of SBS was integrated at 955–975 cm^−1^. The reference bands at 1460 cm^−1^ and 1376 cm^−1^ were integrated at 1445–1475 cm^−1^ and 1365–1390 cm^−1^, respectively. The peak areas were obtained using a local baseline between the two side minima of each selected band.

To investigate the antioxidant effect of lignin, this study evaluated the carbonyl, sulfoxide, and butadiene indices. Two absorption peaks emerged near the conventional 1030 cm^−1^ region. Because previous studies attribute the 1070 cm^−1^ peak to sulfoxide or secondary sulfoxide group formation [41,42], both the 1030 cm^−1^ and 1070 cm^−1^ peaks were selected as thermal-oxidative aging indicators. Furthermore, the polybutadiene index monitors SBS variations across different dosages and aging stages, providing critical evidence to assess lignin’s protective effect on SBS. Although the 1690–1700 cm^−1^ band typically corresponds to carbonyl structures generated during asphalt oxidation, lignin inherently contains carbonyl groups. To prevent absorption interference from lignin incorporation, the carbonyl region was excluded as a comparative aging indicator in this study.

For quantitative comparison, all spectra were normalized using the sum of the integrated areas of the absorption bands at approximately 1460 cm^−1^ (C–H bending vibrations of methylene groups) and 1376 cm^−1^ (symmetric deformation vibrations of methyl groups) as an internal reference. These bands are relatively insensitive to oxidation and aging. The sulfoxide-related aging index and the polybutadiene index were defined as shown in Equations (10) and (11):

(10) $$I_{s=o}=\frac{A_{1030}+A_{1070}}{A_{1460}+A_{1376}}$$

(11) $$I_{PB}=\frac{A_{966}}{A_{1460}+A_{1376}}$$

where *A*_1030_ and *A*_1070_ are the integrated areas of the absorption peaks at 1030 cm^−1^ and 1070 cm^−1^, respectively, and *A*_1460_ and *A*_1376_ are the integrated areas of the reference peaks at 1460 cm^−1^ and 1376 cm^−1^; I_S=O_ is the sulfoxide-related index. I*_PB_* is the polybutadiene index.

## 4. Results and Discussion

### 4.1. Analysis of Molecular Behavior

Prior to conducting MSD and RDF analyses, the simulated density of the model was compared with the measured density of the prepared asphalt binder. As shown in Table 6, the simulated density of the SBS modified asphalt was 1.003 g/cm^3^, whilst the measured value was 1.038 g/cm^3^. Following the addition of lignin, the simulated density increased to 1.006 g/cm^3^, whilst the measured density rose to 1.040 g/cm^3^. Although the simulated density was slightly lower than the measured value, both sets of results exhibited the same increasing trend following the addition of lignin. The discrepancies between the simulation and experimental results may be attributed to the simplified molecular composition of the molecular model, the limited size of the simulation cells, and the idealized mixing state. Similar molecular dynamics studies on SBS modified asphalt have also validated the model by comparing simulated and experimental density ranges, whilst acknowledging that quantitative differences may stem from the model scale and simulation parameters [36]. However, the density increase between the binary and ternary simulation systems was small. Therefore, this result should be interpreted only as weak supporting evidence for a slight change in molecular packing, rather than as proof of a substantially denser structure or a complete microstructural reorganization of the binder.

**Figure 7:**
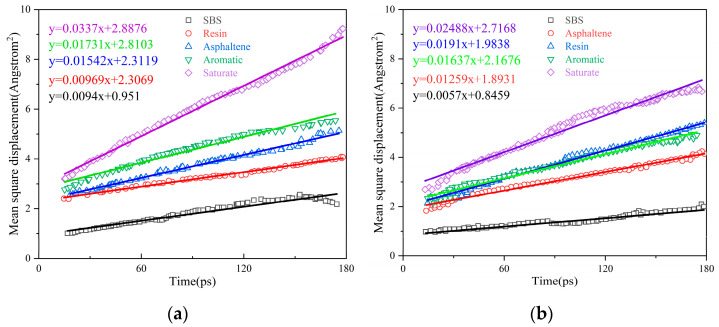
MSD curves of components in asphalt model: (**a**) SBS modified asphalt; (**b**) SBS/lignin composite modified asphalt.

As shown in Figure 7, the MSD curves of all components increased with simulation time in both systems. The diffusion coefficients calculated from the linear fitting of the MSD curves are summarized in Table 7. This result indicates that molecular motion occurred during the simulated period. In the SBS/asphalt system, the slopes of the linear MSD regions followed the order of saturates > aromatics > asphaltenes > resins > SBS. After lignin was introduced, the order changed to saturates > resins > aromatics > asphaltenes > SBS. The slopes for SBS, asphaltenes, aromatics, and saturates decreased, whereas the slope for resins increased. Among these components, SBS showed the most evident decrease in slope. This trend suggests that the simulated Adler lignin model may restrict the mobility of SBS chains and some asphalt fractions in the representative ternary system. Since the slope of the linear MSD region is related to molecular diffusivity, the MSD results indicate that lignin tends to reduce molecular mobility in a component-dependent manner.

**Figure 8:**
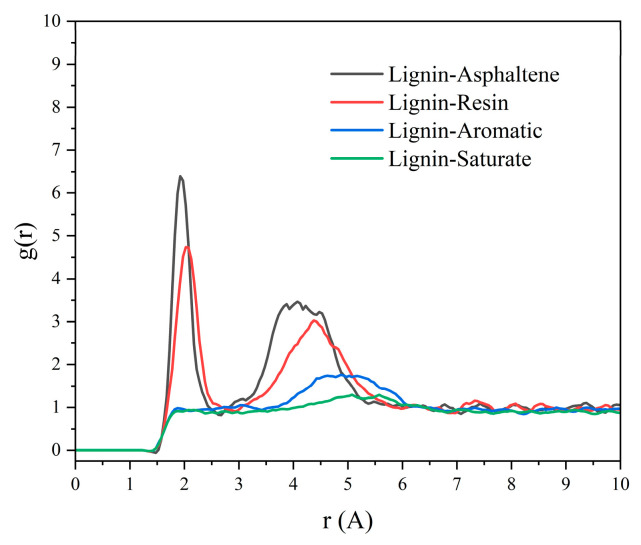
Radial distribution function of lignin and asphalt components.

The RDF results provide further information on the local associations between lignin and asphalt fractions. As shown in Figure 8, the RDF peak intensities followed the order of lignin–asphaltene > lignin–resin > lignin–aromatic > lignin–saturate. The lignin–asphaltene pair showed the highest peak at approximately 1.9–2.0 Å, while the lignin–resin pair showed a weaker peak at approximately 2.0–2.1 Å. In contrast, the lignin–aromatic and lignin–saturate pairs showed broader and weaker peaks mainly between 4 and 5 Å. A short-range RDF peak usually indicates a higher probability of local molecular association. Therefore, these results suggest that the Adler lignin model has a stronger local association tendency with polar and heavier asphalt fractions, especially asphaltenes and resins, than for weakly polar saturates.

Taken together, the density, MSD, and RDF results suggest that lignin is not completely randomly distributed in the SBS modified asphalt system. Instead, it tends to associate with the asphaltene- and resin-rich fractions. The small increase in equilibrium density from 1.003 to 1.006 g/cm^3^ only indicates a slight change in molecular packing and should be regarded as supporting evidence rather than direct proof of a more compact structure. The MSD results show that lignin addition reduced the mobility of SBS and most asphalt fractions, while the RDF results indicate stronger local association with asphaltenes and resins. These findings suggest that lignin may influence the microstructural organization of SBS-modified asphalt by interacting preferentially with heavy polar fractions and by limiting molecular diffusion to some extent.

It should be noted that the molecular dynamics simulation in this study was conducted using one representative SBS/lignin/asphalt ternary model. Therefore, the simulation results mainly provide molecular-level evidence for the interaction between lignin and asphalt components, the enhancement of molecular packing, and the restriction of molecular diffusion. However, the model cannot fully account for the effects of varying lignin content on the rutting resistance and fatigue life of modified asphalt.

### 4.2. Temperature Sweep Results

**Figure 9:**
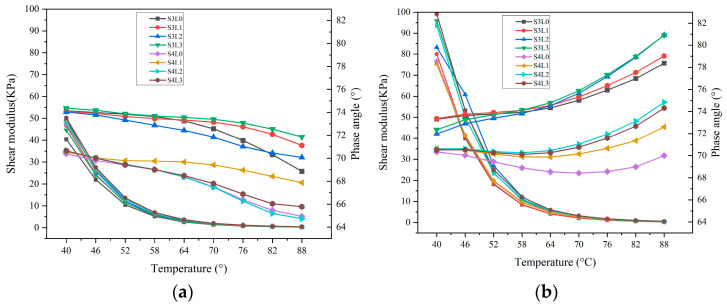
Modulus and phase angle of SBS/lignin composite modified asphalt: (**a**) virgin asphalt and (**b**) RTFO asphalt.

The phase angle represents the proportion of elastic versus viscous components in a material. As shown in Figure 9, with increasing temperature, the phase angle of the aged samples first decreases and then increases. This is a unique rheological characteristic of SBS-modified asphalt, reflecting the process of free asphalt loss, exposure of structured asphalt, and subsequent softening and failure of the structured asphalt. Further analysis of the effect of lignin on the phase angle of SBS-modified asphalt reveals that as the lignin content increases, the phase angle of the modified asphalt further decreases, and this effect is more developed at high temperatures. This indicates that lignin acts as an elastic component in SBS-modified asphalt, enhancing the overall elastic response of the asphalt binder.

The complex modulus reflects the high-temperature stiffness of a material. Figure 9a,b shows the complex modulus (G*) curves of SBS/lignin composite modified asphalts before and after short-term aging. The results indicate that G* of all samples decreases monotonically with increasing temperature, which follows the viscoelastic behavior of asphalt materials. In the temperature range of 40–60 °C, asphalt binders with high modifier contents (e.g., 4S3L) exhibit higher G* values than those with low modifier contents (e.g., 3S, 3S1L), indicating that the total amount of modifiers tends to enhance the high temperature stiffness of asphalt. In the high temperature range of 70–88 °C, for modified asphalts with the same SBS content, G^*^ increases with increasing lignin content, indicating that the introduction of lignin can enhance the overall high-temperature stiffness.

**Figure 10:**
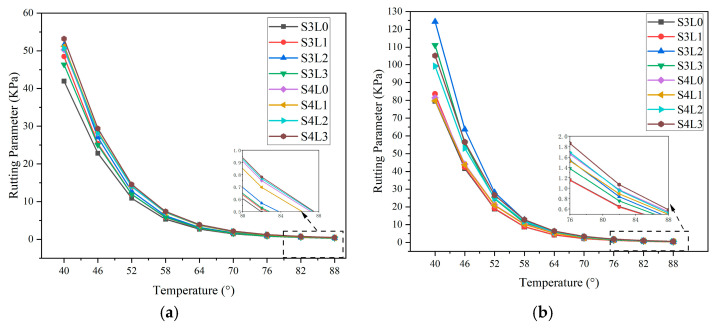
Rutting parameter of SBS/lignin composite modified asphalt: (**a**) Virgin asphalt and (**b**) RTFO asphalt.

The rutting factor directly reflects the deformation resistance of a material. Figure 10 shows the temperature dependence of the rutting factor (G*/sin *δ*) for each SBS/lignin composite modified asphalt. Similar to the trend observed in Figure 10, an increase in temperature leads to a rapid decrease in G*/sin *δ*, indicating that high temperature weakens the physical crosslinking within the asphalt, thereby reducing its deformation resistance. Further analysis of the effect of different blending ratios reveals that the introduction of lignin increases the rutting factor. At the same SBS content, increasing the lignin content results in a higher G*/sin *δ* in the high-temperature range, indicating improved resistance to shear deformation.

As shown in Figure 10a,b, after short-term aging, the G*and G*/sin *δ* values of all samples were generally higher than those of the corresponding virgin binders, indicating that RTFO treatment caused a certain degree of hardening in the asphalt system and thereby enhanced its resistance to permanent deformation at high temperature. The lignin-modified groups still maintained higher modulus and rutting factor values than the corresponding SBS control group after short-term aging, and the relative ranking among different formulations remained essentially unchanged. This suggests that the high-temperature reinforcing effect of lignin is reasonably stable and does not disappear under short-term thermo-oxidative aging.

From the combined analysis of lignin’s effects on the phase angle (*δ*), complex modulus (G*), and rutting factor (G*/sin δ) of SBS modified asphalt, the results suggest that lignin enhances the deformation resistance of SBS modified asphalt by introducing rigid particles, which increase the elastic component and improve the high-temperature stiffness.

### 4.3. Multiple Stress Creep Recovery Test Analysis

**Figure 11:**
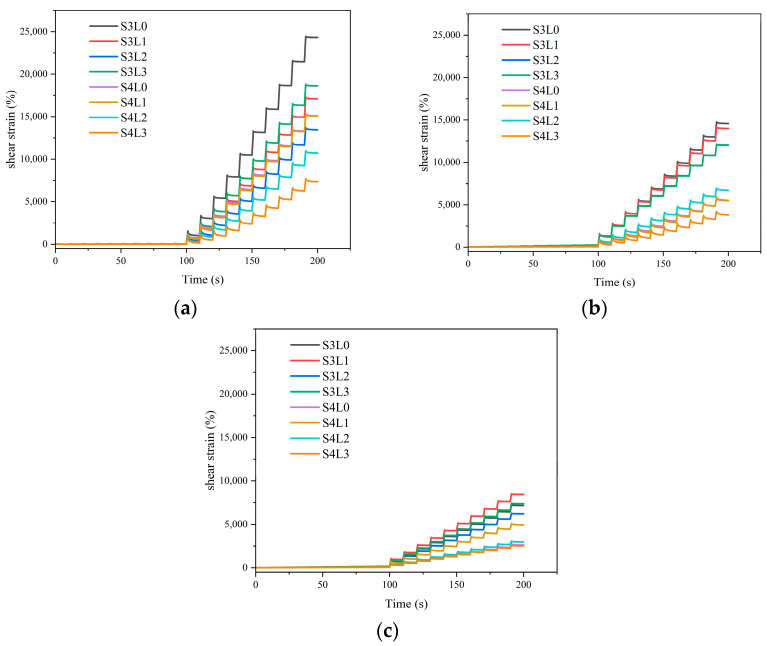
Shear strain curves of SBS/lignin composite modified asphalt: (**a**) virgin asphalt, (**b**) RTFO asphalt, (**c**) PAV asphalt.

Figure 11a–c shows the cumulative shear strain curves over time for SBS/lignin composite modified asphalts under different aging conditions, reflecting the effect of lignin on the nonlinear viscoelastic response of SBS-modified asphalt. From the overall trend observed in the three figures, the cumulative shear strain of the samples gradually decreases with increasing aging degree, indicating that aging hardens the asphalt, making it difficult to undergo large deformation under the same loading conditions.

Notably, after short-term aging (RTFO) and long-term aging (PAV), the shear strain curves of the samples exhibit a layered trend. The curves form distinct layers based on SBS content: the 4% SBS samples lie at the bottom and the 3% SBS samples at the top. This phenomenon indicates that SBS is the dominant factor determining the high-temperature rutting resistance of SBS/lignin composite modified asphalt.

At the same time, the improvement effect of lignin on the asphalt binder should not be overlooked. At the same SBS content, increasing the lignin content causes the shear strain curves to shift downward in a regular manner. This observation indicates that although the introduction of lignin does not alter the SBS-dominated network structure, it helps reduce the internal fluidity of the asphalt binder and suppresses viscous flow at high temperatures.

**Figure 12:**
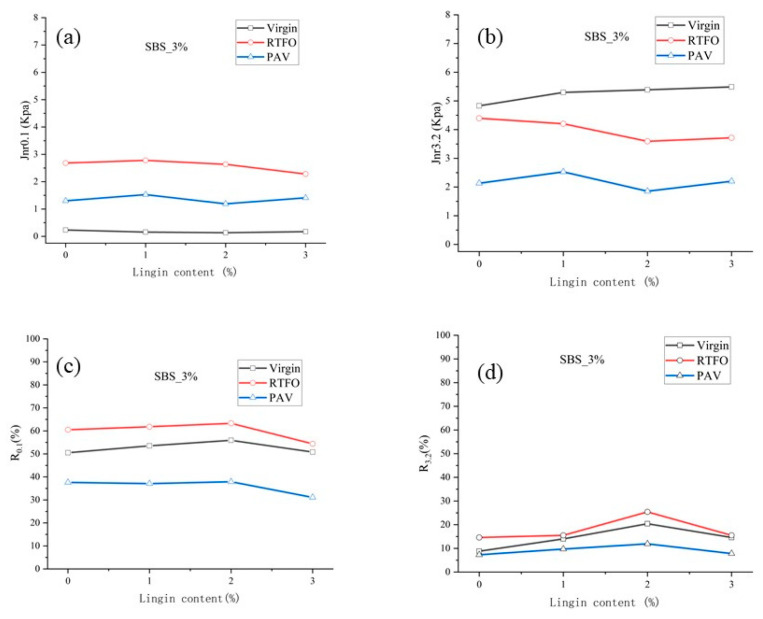
Effects of lignin content and aging on the MSCR parameters of 3% SBS modified asphalt: (**a**) Jnr at 0.1 kPa; (**b**) Jnr at 3.2 kPa; (**c**) R at 0.1 kPa; (**d**) R at 3.2 kPa.

**Figure 13:**
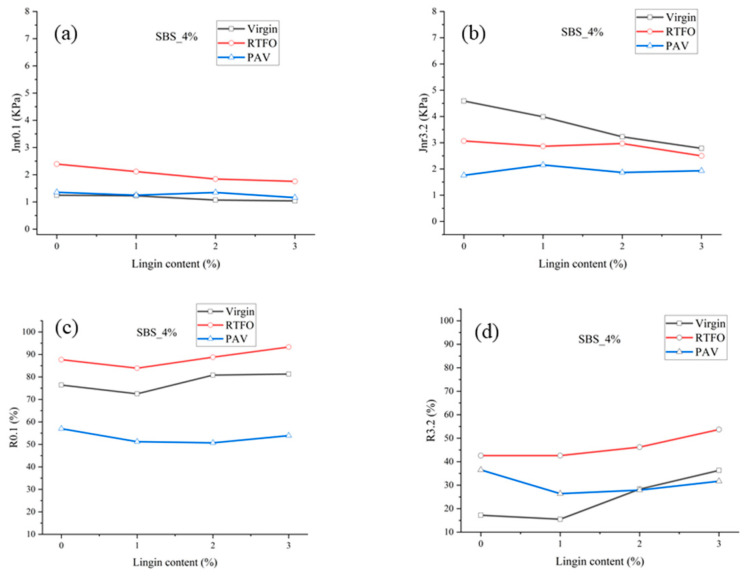
Effects of lignin content and aging on the MSCR parameters of 4% SBS modified asphalt: (**a**) Jnr at 0.1 kPa; (**b**) Jnr at 3.2 kPa; (**c**) R at 0.1 kPa; (**d**) R at 3.2 kPa.

To quantitatively analyze the effect of lignin on the elastic recovery performance of SBS-modified asphalt, this study calculated the non-recoverable creep compliance (J_nr_) and elastic recovery rate (R) exhibited by each sample in the MSCR test. The results are shown in Figure 12 and Figure 13.

Since 0.1 kPa falls within the low-stress linear viscoelastic region, the properties reflected at this stress level are consistent with those from temperature sweep tests. Moreover, all materials exhibit relatively low non-recoverable creep and high elastic recovery rates, offering little differentiation. Therefore, this analysis focuses primarily on R and J_nr_ at 3.2 kPa.

As shown in Figure 12 and Figure 13, the elastic recovery rate of the asphalt binder exhibits a non-monotonic trend with the aging process: it first increases during the short-term aging stage and then decreases rapidly after long-term aging. This phenomenon can be attributed to the secondary development behavior of SBS in the asphalt matrix. In this study, the preparation conditions of the SBS/lignin composite modified asphalt were relatively mild and the development time was short, resulting in an incompletely formed SBS network structure. Under these conditions, the thermo-oxidative effect of the RTFO test promoted further crosslinking and growth of the SBS network, thereby enhancing the elastic recovery capability. However, the harsh conditions of the PAV test caused damage to the already formed polymer network, leading to a reduction in material elasticity.

Comparing the two systems, the 4% SBS formulation generally exhibited lower Jnr and higher R values across all aging states, particularly at 3.2 kPa. Based on the combined analysis of the shear-strain curves, non-recoverable creep compliance, and elastic recovery rate from the MSCR test, lignin has a certain influence on the high temperature deformation resistance of SBS modified asphalt, but SBS remains the dominant factor determining the final performance of the modified asphalt.

### 4.4. LAS Fatigue Performance Results and Discussion

**Figure 14:**
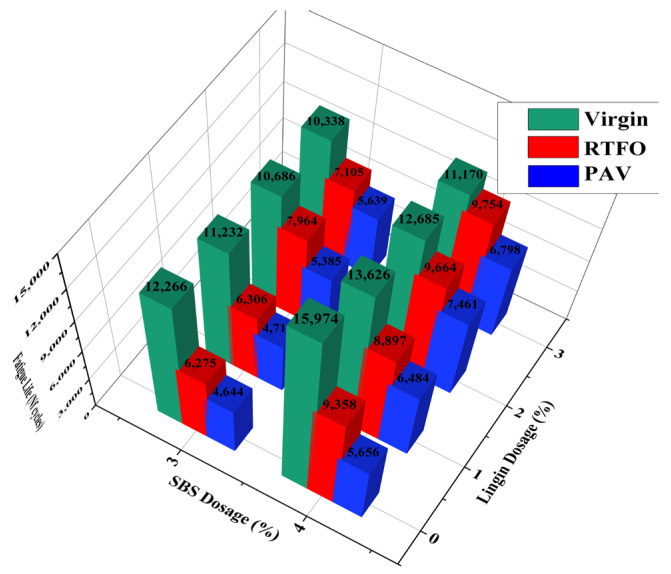
Fatigue life at 2.5% strain for SBS/lignin composite modified asphalt under different aging conditions.

**Figure 15:**
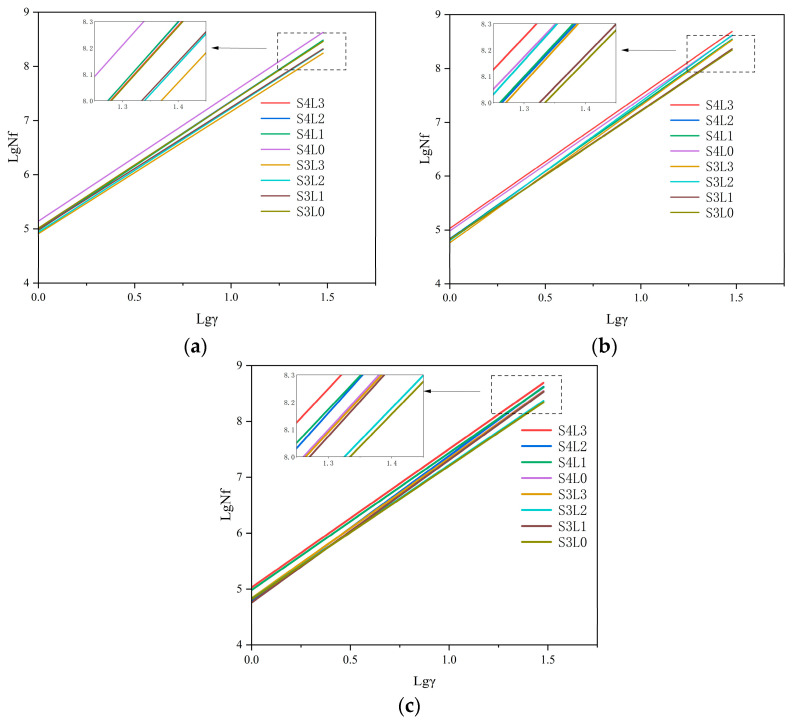
Fatigue life curves of SBS/lignin composite modified asphalt (**a**) Virgin asphalt (**b**) RTFO asphalt (**c**) PAV asphalt.

Figure 14 shows the fatigue life at 2.5% strain of SBS/lignin composite modified asphalts under different aging conditions and Figure 15 shows the fatigue life curves of SBS/lignin composite modified asphalts. The results indicate that SBS content is the dominant factor determining the fatigue life of the material. Furthermore, it can be seen from the figure that in the unaged state, the fatigue curve of the asphalt without lignin lies above that with lignin. However, after short-term and long-term aging, the curve for the asphalt containing 3% lignin and 4% SBS is located at the top. This suggests that the introduction of lignin exhibits a brittle effect on SBS-modified asphalt in the unaged state. During the unaged stage, lignin, acting as rigid polymer particles dispersed within the SBS network structure, restricts the free deformation and relaxation of SBS segments under cyclic loading, suppresses energy dissipation in the asphalt binder under stress, and makes internal microcracks more likely to initiate and propagate, thereby reducing its fatigue life. Moreover, this embrittling effect intensifies with increasing lignin content. Under short-term and long-term aging conditions, lignin may play a protective role in SBS modified asphalt. By exerting antioxidant effects through active functional groups such as phenolic hydroxyl groups, it inhibits the aging degradation of SBS to a certain extent, thereby maintaining the toughness of the material under thermo-oxidative aging. As a result, the groups containing lignin exhibit a higher fatigue life under both short-term and long-term aging conditions.

This reversal in the effect of lignin on fatigue life from the unaged to the aged state can be explained by the competing influences of rigid-filler embrittlement and radical-scavenging antioxidant protection. In the unaged binder, lignin particles act as rigid inclusions within the SBS-asphalt matrix. They restrict the mobility and relaxation of SBS segments under cyclic loading, inhibit energy dissipation, and promote local stress concentrations that accelerate microcrack initiation and propagation, thereby reducing fatigue life. Under thermo-oxidative aging, however, the dominant degradation mechanism is the oxidative chain scission and crosslinking of the polybutadiene segments of SBS. Lignin, through its phenolic hydroxyl groups, may donate active hydrogen atoms to quench free radicals, thereby interrupting the oxidative chain reaction and protecting the SBS network. This preservation of the elastomeric phase counteracts the stiffening effect, and the retained network flexibility ultimately leads to a higher fatigue life than that of the aged SBS-only control. The highest PAV-aged fatigue life observed for S4L3 (4% SBS + 3% lignin) suggests that the antioxidant benefit outweighs the intrinsic embrittlement when the binder is subjected to severe long-term aging, which is precisely the condition where oxidative degradation is most critical.

### 4.5. Bending Beam Rheometer Results Analysis and Discussion

**Figure 16:**
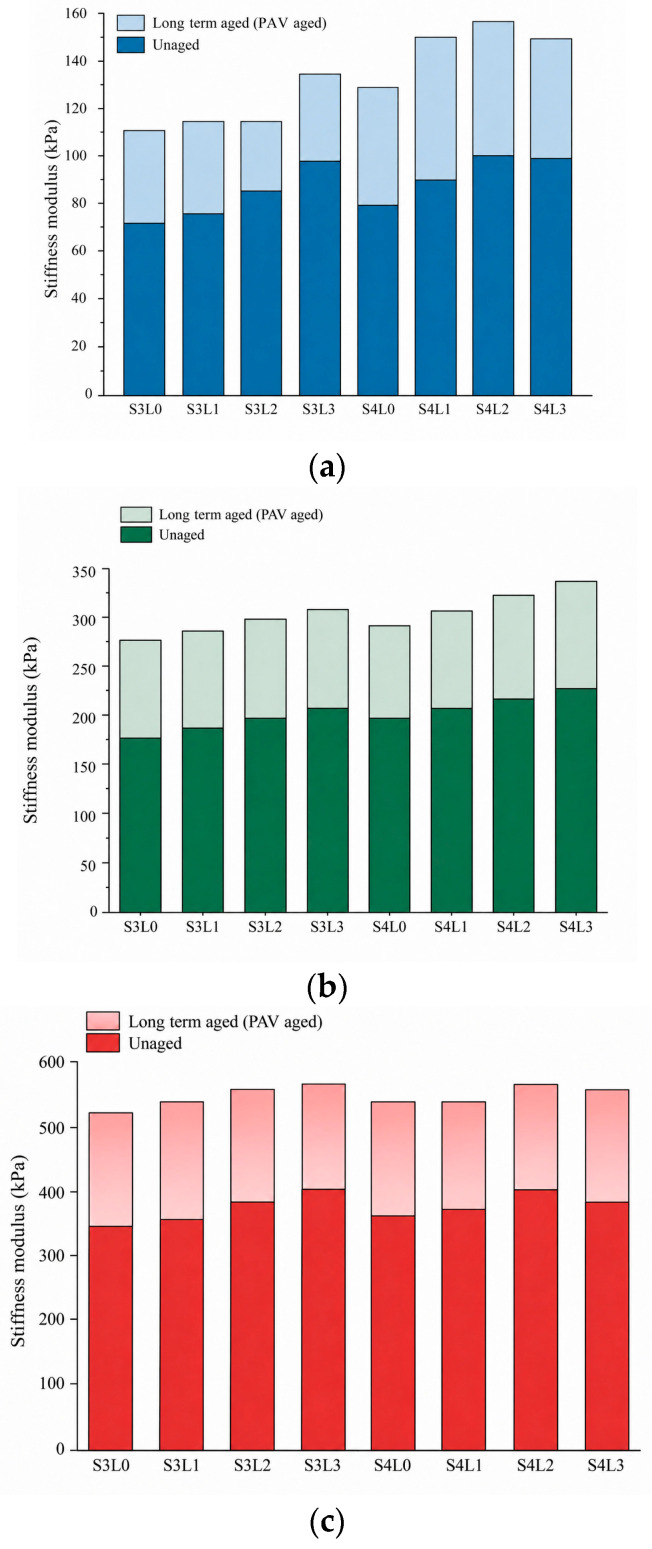
Stiffness modulus of SBS/lignin composite modified asphalt: (**a**) stiffness modulus at −6 °C; (**b**) stiffness modulus at −12 °C; (**c**) stiffness modulus at −18 °C.

Figure 16 shows the stiffness modulus of SBS/lignin composite modified asphalt at −6 °C, −12 °C, and −18 °C, reflecting the effect of lignin on the low-temperature stiffness of SBS-modified asphalt. As shown in Figure 16, the stiffness modulus of asphalt binders increased markedly as the test temperature decreased. This trend is consistent with the rheological behavior of asphalt, which gradually changes from a viscoelastic material to a more elastic material at lower temperatures. Increasing the SBS content also increased the stiffness modulus at all three temperatures. This can be attributed to the reinforcing effect of the polymer network formed by SBS, which acts as a physical skeleton and restricts the free movement of asphalt molecules, thereby increasing the low-temperature stiffness of the material.

The effect of lignin on the stiffness modulus of SBS-modified asphalt indicates that lignin further enhances the low-temperature stiffness of the binder. In the unaged state, the stiffness modulus of SBS-modified asphalt containing lignin was higher than that of the SBS-only modified asphalt at all three test temperatures, and it increased with increasing lignin content. Long-term aging by PAV also increased the stiffness modulus of the asphalt binders, which was mainly caused by the hardening and embrittlement of the asphalt matrix during aging. By comparing the aging-induced increments among different samples, it can be found that the increase in stiffness modulus of the lignin-containing groups was smaller than that of the SBS-modified asphalt control group. This suggests that lignin can inhibit, to some extent, the aging-induced deterioration of the low-temperature performance of SBS-modified asphalt.

**Figure 17:**
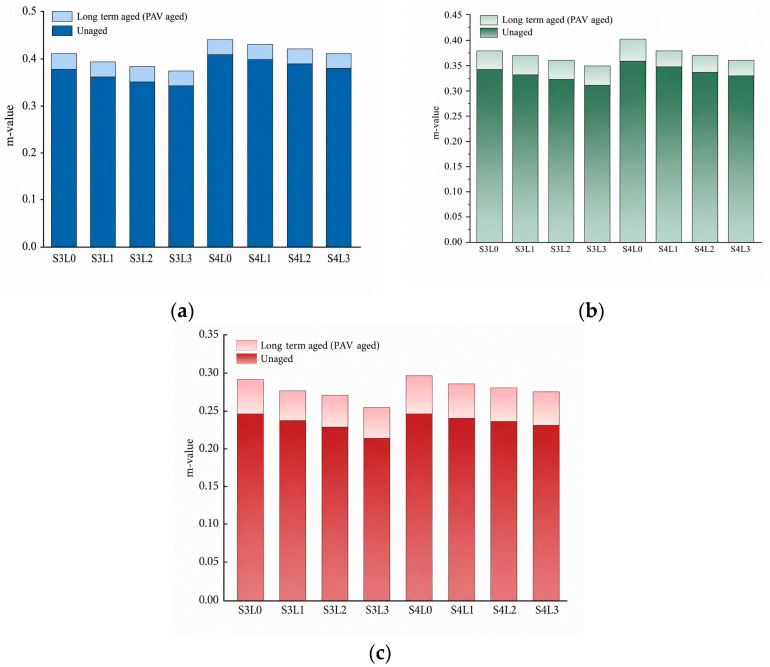
M-value of SBS/lignin composite modified asphalt: (**a**) m-value at −6 °C; (**b**) m-value at −12 °C; (**c**) m-value at −18 °C.

Figure 17 presents the creep rate of SBS/lignin composite modified asphalt at the three test temperatures, reflecting the effects of lignin and SBS on the stress relaxation capacity of the asphalt matrix. The incorporation of SBS improved the low-temperature stress relaxation capacity of the asphalt matrix. As the SBS content increased, the creep rate of the modified asphalt showed an upward trend. This is because SBS can form a network structure in the asphalt matrix, which provides stronger elastic recovery capacity and improves the stress relaxation performance of the material.

In contrast, the incorporation of lignin weakened the low-temperature stress relaxation capacity of the asphalt matrix. At a fixed SBS content, the creep rate of the asphalt binder decreased with increasing lignin content. This can be attributed to the fact that lignin increases the elastic component of the asphalt material, causing the overall viscoelastic behavior of the binder to shift toward a more elastic response and thereby reducing its stress relaxation capacity.

A comparison of the creep rates before and after aging shows that all samples exhibited a decreasing trend after aging, indicating that thermo-oxidative aging also deteriorated the low-temperature stress relaxation capacity of asphalt binders. Further analysis shows that the introduction of lignin can mitigate the aging-induced reduction in creep rate. In samples with higher lignin contents, such as 4S3L and 3S3L, the decrease in creep rate caused by aging was lower than that of the SBS modified asphalt control group. This phenomenon is consistent with the trend observed in the stiffness modulus analysis, both indicating that lignin can enhance the resistance of SBS modified asphalt to thermo-oxidative aging and slow down the aging-induced deterioration of low-temperature performance.

### 4.6. FTIR Data Analysis and Discussion

**Figure 18:**
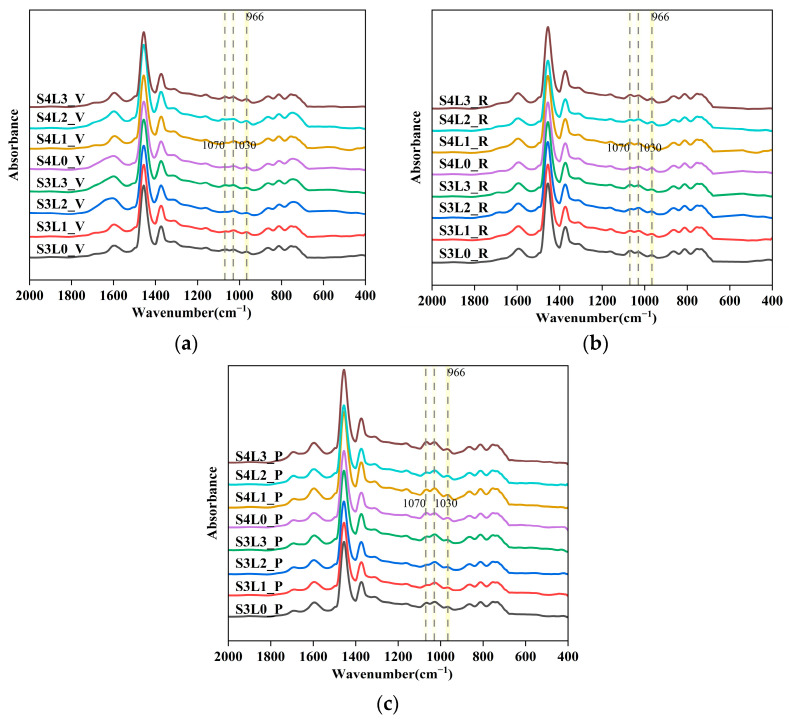
FTIR spectra of SBS/lignin composite modified asphalt: (**a**) Virgin asphalt; (**b**) RTFO asphalt; (**c**) PAV asphalt.

As shown in Figure 18, the principal absorption peak positions of the SBS/lignin composite-modified asphalt remained stable across the virgin, short-term (RTFO), and long-term (PAV) aging states, whereas the peak intensities evolved progressively. Furthermore, the absence of new characteristic absorption peaks upon lignin incorporation indicates that lignin does not appear to induce new covalent reactions detectable by FTIR within the system.

**Figure 19:**
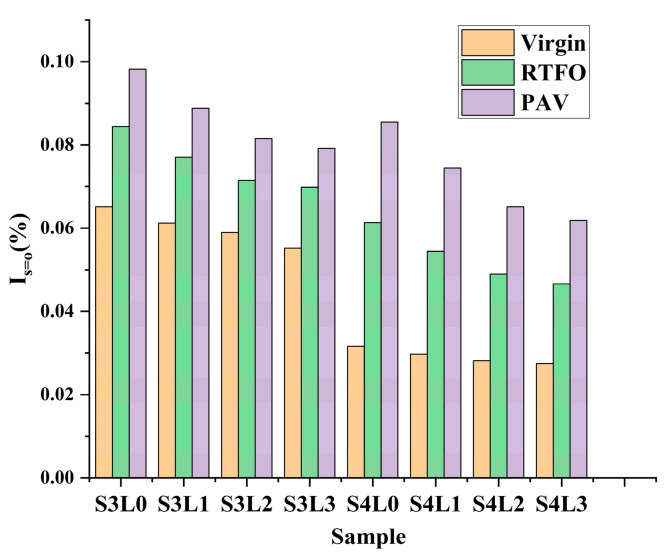
Effect of aging states and modifier dosages on the sulfoxide-related aging index (Is = o) of SBS/lignin composite modified asphalt.

**Figure 20:**
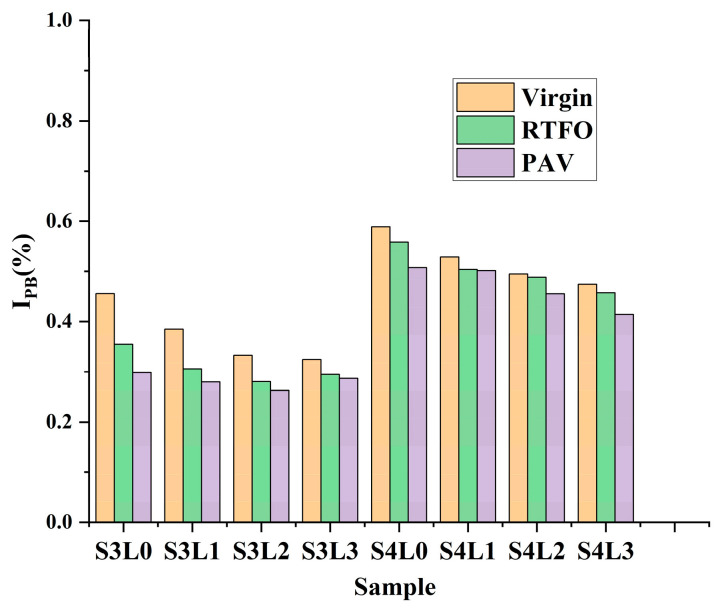
Effect of aging states and modifier dosages on the polybutadiene index (I*_PB_*) of SBS/lignin composite modified asphalt.

As shown in Figure 19 and Figure 20, the sulfoxide-related aging index increased with aging, whereas the polybutadiene index decreased. This indicates that thermal-oxidative aging promoted the accumulation of sulfur-related oxidation products and the degradation of the SBS polybutadiene segments. At the same SBS dosage, lignin-containing binders generally showed a lower increase in the sulfoxide-related aging index and a smaller decrease in the polybutadiene index than the SBS-only binders. These results suggest that lignin may slow the oxidative aging of the asphalt matrix and reduce the degradation of SBS to some extent.

It should be noted that the absorption bands around 1030 cm^−1^ and 1070 cm^−1^ may also be affected by oxygen-containing structures in lignin and other asphalt components. Therefore, the sulfoxide-related aging index used in this study should be interpreted as a relative aging indicator rather than a direct quantitative measurement of sulfoxide concentration. Similarly, the FTIR results alone cannot fully prove the radical-scavenging pathway of lignin. Instead, the observed FTIR trends provide supporting evidence for the anti-aging effect of lignin when considered together with the rheological aging results.

### 4.7. Comprehensive Performance Evaluation

The present results should be interpreted within the evidence boundary of a binder-scale study. The MD results suggest local association tendencies between lignin and polar asphalt fractions and component-dependent mobility changes, while the rheological and FTIR results indicate improved high-temperature stiffness, modified fatigue response, and slower evolution of oxidation-related spectral indices. However, these findings do not directly establish a fully resolved composite morphology, long-term dispersion stability, or pavement-scale durability. Recent studies on asphalt interfacial modification have shown that claims regarding interfacial reinforcement or nanostructure regulation are usually supported by multiple techniques, such as molecular simulation, surface thermodynamic analysis, interfacial mechanical testing, and microstructural characterization [43]. Therefore, the present MD results are used as qualitative mechanistic support rather than direct morphological proof. Similarly, the fatigue results should be discussed separately from aging resistance. Lignin increased stiffness and reduced fatigue life in the unaged state, whereas the aged binders showed improved fatigue retention. This trade-off indicates that thermal-oxidative aging resistance and fatigue tolerance are coupled but not identical performance dimensions. Recent studies on thermal-fatigue and high-temperature degradation in construction composites also emphasize the need to separate thermal damage, fatigue response, microstructural evolution, and service durability [44]. Accordingly, the present study supports the potential of lignin as an auxiliary antioxidant modifier for SBS-modified asphalt at the binder scale, but further storage stability, fluorescence microscopy, AFM phase imaging, viscosity evolution, mixture rutting, mixture fatigue, moisture damage, and field validation are still needed.

To compare the relative performance of the tested binders, the eight formulations were evaluated in terms of high-temperature deformation resistance, fatigue response, and aging-related indicators. The results show that the SBS dosage largely controls the basic elastic network. The 4% SBS series generally shows better rheological recovery than the 3% SBS series. Within the S4Lx group, adding lignin creates a clear trade-off. On the one hand, lignin clearly improves high temperature deformation resistance (higher rutting factor, lower Jnr) and reduced non-recoverable deformation during aging, as shown by FTIR and the aged LAS results. On the other hand, too much lignin—as in the S4L3 formulation—makes the binder excessively stiff. This stiffness limits the material’s ability to relax and, in turn, lowers its initial fatigue life in the unaged state. Within the tested formulation range, lignin addition improved high-temperature deformation resistance and reduced the aging-related changes indicated by FTIR and aged LAS results. These trends are consistent with a possible oxidation-retarding contribution of lignin. However, the exact antioxidant pathway, including any radical-scavenging process, cannot be directly confirmed by the present molecular model or FTIR results alone. Therefore, S4L2 is regarded as a balanced formulation within the tested binder matrix, rather than as a generally optimized SBS/lignin asphalt system.

## 5. Conclusions

This study used a molecular dynamics simulation and laboratory experiments to investigate the rheological and antioxidative properties of styrene-butadiene-styrene (SBS)/lignin composite modified asphalt. The main conclusions are as follows.

1. The molecular dynamics results based on the Adler lignin model suggested possible local association tendencies between lignin and heavy polar asphalt fractions, especially asphaltenes and resins. These results provide qualitative molecular-level support for local lignin–asphalt interactions, but they should not be interpreted as direct confirmation of the exact chemistry of the experimental corncob enzymatic hydrolysis lignin.
2. Lignin addition changed the physical and rheological response of SBS-modified asphalt mainly through a stiffening and filler-reinforcing effect. In the rheological tests, lignin reduced the phase angle and increased the complex modulus, rutting factor, and elastic contribution, indicating improved resistance to high-temperature deformation at the binder scale.
3. The LAS results showed that lignin reduced the fatigue life of SBS-modified asphalt in the unaged state, mainly due to increased stiffness and stress concentration. After long-term aging, the lignin-containing binders showed better fatigue retention than the SBS-only control. This result suggests a possible aging-retarding contribution of lignin, but the lignin dosage should be carefully controlled to balance the stiffening effect and the aging-related benefit.
4. The FTIR results showed that lignin-containing binders exhibited lower sulfoxide-related indices and a slower reduction in the polybutadiene-related index during aging. These results provide semi-quantitative evidence for reduced oxidation-related functional group evolution and better retention of SBS-related spectral features.
5. Within the investigated SBS/lignin dosage range, S4L2 showed a relatively balanced performance among high-temperature deformation resistance, aging-related indicators, and fatigue retention. Therefore, S4L2 can be regarded as a favorable formulation in the tested binder matrix. However, because the experimental design only included 3% and 4% SBS dosages and did not include lignin-only modified asphalt as a control, this result should not be interpreted as a generally optimized SBS/lignin asphalt formulation.

Several limitations should be acknowledged. The molecular dynamics simulation was based on one representative SBS/lignin/asphalt ternary system and used an idealized Adler softwood lignin model, which cannot fully represent the source-specific chemistry of the corncob enzymatic hydrolysis lignin used in the laboratory. Moreover, the classical force field cannot describe oxidation reactions, bond dissociation, or free-radical evolution. Therefore, the MD results provide only qualitative molecular-level support for possible local association patterns and component-dependent mobility changes, rather than direct evidence of the actual morphology, dispersion state, or antioxidant reaction pathway of the experimental binder. The FTIR results should also be interpreted as semi-quantitative indicators of oxidation-related functional group evolution, not as direct proof of a complete antioxidant mechanism or SBS molecular protection pathway. In addition, the formulation design was limited to the selected SBS/lignin dosage matrix and did not include lignin-only controls or broader modifier contents. Thus, S4L2 should be regarded only as a favorable formulation within the investigated binder matrix, rather than as a generally optimized SBS/lignin asphalt system. Although BBR testing strengthened the binder-scale evaluation, storage stability, fluorescence microscopy, AFM phase imaging, viscosity evolution, mixture-scale rutting, mixture fatigue, moisture damage evaluation, and field validation were not included. Therefore, the present results support the potential of lignin for improving binder-scale aging resistance and rheological performance, but they do not verify pavement-scale durability. In addition, although lignin is renewable and may reduce partial reliance on petroleum-based modifiers, this study did not include life-cycle assessment, cost analysis, preparation-emission evaluation, supply-chain variability assessment, mixing-energy analysis, or long-term service evidence. Thus, the sustainability of SBS/lignin composite modified asphalt should be regarded as a potential advantage that requires further environmental and economic verification, rather than as a demonstrated outcome of the present study. Future work should combine refined molecular modeling, source-specific lignin characterization, broader formulation testing, microstructural validation, mixture-level performance evaluation, field trials, and life-cycle-based sustainability assessment.

## Figures and Tables

**Table 1:** Physical properties of 70# asphalt.

Item	Unit	Result	Standard
Penetration (25 °C)	0.1 mm	64	ASTM D5
Penetration Index (25 °C)		−1.25	ASTM D5
Soften Point	°C	48	ASTM D36
Ductility (15 °C)	cm	>100	ASTM D113
Ductility (10 °C)	cm	28	ASTM D113
Rotational Viscosity (135 °C)	mPa·s	370	ASTM D4402
Density (15 °C)	g/cm^3^	1.036	ASTM D70

**Table 2:** Physical Properties of SBS.

Physical Properties	Unit	Result	Standard
Specific Gravity	g/cm^3^	0.94	ASTM D792
Ash Content	%	0.3	ASTM D5667
Volatile Matter Content	%	0.25	ASTM D5668
Styrene Content	%	31	ASTM D5775

**Table 3:** Physical properties of lignin.

Physical Properties	Unit	Result
Effective lignin content	%	70
Cellulose content	%	23
Hemicellulose content	%	5
Ash content	%	2
Particle size	μm	30
Weight-average molecular weight	g/mol	2037
Number-average molecular weight	g/mol	1179

**Table 4:** Binder codes and modifier contents.

Codes of Binders	SBS Content (%)	Lignin Content (%)
S3L0	3	0
S3L1	3	1
S3L2	3	2
S3L3	3	3
S4L0	4	0
S4L1	4	1
S4L2	4	2
S4L3	4	3

**Table 5:** Detailed information of the models used in the molecular simulation.

Material	Molecular	Chemical Formulas	Numbers in Simulation Cells
Lignin	Adler	C160H180O58	1
SBS	SBS	C260H352	1
Asphalt	Phenol	C42H54O	3
	Thiophene	C51H62S	3
	Pyrrole	C66H81N	2
	Pyridinohopane	C36H57N	4
	Quinolinohopane	C40H59N	4
	Thioisorenieratane	C40H60S	4
	Benzobisbenzothiophe	C18H10S2	15
	Trimethylbenzeneoxane	C29H50O	5
	PHPN	C35H44	11
	DOCHN	C30H46	13
	Squalane	C30H62	4
	Hopane	C35H62	4

**Table 6:** Comparison between simulated and measured densities.

Binder	Simulated Density (g/cm^3^)	Measured Density (g/cm^3^)
SBS modified asphalt	1.003	1.038
SBS/lignin composite modified asphalt	1.006	1.040

**Table 7:** Diffusion coefficients calculated from the linear fitting of MSD curves.

System	Component	*R* ^2^	D (10^−11^ m^2^/s)	SE of D
SBS/asphalt	Saturates	0.99257	5.62	0.053
SBS/asphalt	Aromatics	0.96755	2.89	0.057
SBS/asphalt	Asphaltenes	0.99015	2.57	0.029
SBS/asphalt	Resins	0.99186	1.62	0.019
SBS/asphalt	SBS	0.9364	1.57	0.045
SBS/lignin/asphalt	Saturates	0.98128	4.15	0.047
SBS/lignin/asphalt	Resins	0.99305	3.18	0.029
SBS/lignin/asphalt	Aromatics	0.9835	2.73	0.034
SBS/lignin/asphalt	Asphaltenes	0.98718	2.1	0.027
SBS/lignin/asphalt	SBS	0.90591	0.95	-

The unit conversion 1 Å^2^/ps = 10^−8^ m^2^/s was applied. The SE values represent the standard error of the linear fitting slope.

## Data Availability

Data will be made available on request.
